# Digital Analysis of Subtrochlear Sclerosis in Elbows Submitted for Dysplasia Screening

**DOI:** 10.3389/fvets.2021.664532

**Published:** 2021-05-12

**Authors:** Ana Válega, Sofia Alves-Pimenta, Fintan J. McEvoy, Dorte H. Nielsen, Mário Ginja

**Affiliations:** ^1^Department of Veterinary Science, University of Trás-os-Montes and Alto Douro, Vila Real, Portugal; ^2^Department of Animal Science, University of Trás-os-Montes and Alto Douro, Vila Real, Portugal; ^3^CITAB—Centre for the Research and Technology of Agro-Environmental and Biological Sciences, University of Trás-os-Montes and Alto Douro, Vila Real, Portugal; ^4^Department of Veterinary Clinical Sciences, Faculty of Health and Medical Sciences, University of Copenhagen, Copenhagen, Denmark

**Keywords:** elbow dysplasia, screening, breeding, trabecular pattern, medial coronoid disease

## Abstract

Ulnar trochlear notch (UTN) subchondral bone sclerosis is observed in elbow dysplasia (ED) associated with the medial coronoid disease. However, its evaluation is based on a simple visual examiner assessment of bone radio-opacity level and is considered subjective. The purpose of this study was to objectively characterize the radiographic opacity of the ulnar trochlear notch (UTN) subchondral bone in mediolateral elbow projections classified, using the International Elbow Working Group guidelines. Records and mediolateral flexed elbow images from the Danish Kennel Club database for the ED screening scheme between 2012 and 2018 were available. Of the dogs in the database, those with an ED-negative status in the left limb were identified. From these, 20 dogs each having a status free from ED, or with Grade 1, 2, or 3 in the right limb, were randomly chosen. Joints with primary ununited anconeal process were excluded from the sample. A template was developed using the ImageJ software, for computer UTN sclerosis analysis. It was overlaid onto each image to define five regions of interest (ROIs): ROI-1, distal UTN; ROI-2, middle UTN; ROI-3, caudal UTN; ROI-4, cortical bone; and ROI-5, bone marrow. Mean pixel intensity for each UTN ROI was divided by the mean pixel intensity of ROI-4 to normalize the data. The mean ± standard deviation (SD) of the normalized pixel intensity in the disease joints (ED Grades 1, 2, and 3) was 1.18 ± 0.17, 1.03 ± 0.12, and 0.92 ± 0.09 for ROIs 1, 2, and 3, respectively. The corresponding values for the contralateral normal left joints were 1.16 ± 0.17, 1.01 ± 0.1, and 0.91 ± 0.08. There was a significant difference (*P* < 0.05) in the normalized mean pixel intensity in dysplastic vs. non-dysplastic elbow joints for ROIs 1 and 2. The raw mean pixel intensity from right and left cortical and marrow bone ROIs sometimes showed relatively large differences. Digital radiography is associated with exposure and post-processing variabilities. Differences in apparent radio-opacity (as indicated by pixel intensity) though statistically significant in dysplastic joints compared with contralateral normal joints are slight and are thus problematic for computer-aided assessments of UTN sclerosis.

## Introduction

Elbow dysplasia (ED) in dogs is a developmental hereditary disease, which includes one or more of these primary joint conditions: ununited anconeal process (UAP), osteochondritis dissecans (OCD) of the humeral condyle, medial coronoid disease (MCD), and incongruency of the humero-ulnar joint (1, 2). These conditions result in secondary osteoarthritis leading to pain, discomfort, and lameness (3). The incidence of ED depends on the breed, the population, the screening technique, and the database source, but affected animals can reach up to 70% in some populations (4).

The treatment options for ED are considered relatively limited, and the International Elbow Working Group (IEWG) founded in 1989 recommends radiographic screening of the elbow joint so that dogs with better joint conformation can be selected for breeding. The aim of these schemes is to gradually reduce the prevalence of the disease in canine populations (5). Over the years, the IEWG has developed radiographic diagnostic protocols and scoring system guidelines, which are regularly updated. The IEWG and their guidelines are considered as the international reference among the scientific community for ED screening in most countries concerned with the problem (5–7). However, there are some divergences among national organizations with respect to the type of radiographic projections required for ED scoring. One or more of the following elbow projections may be required: flexed mediolateral, neutral or extended mediolateral, and craniocaudal (or craniocaudal with 15° of pronation). The IEWG ED scoring guidelines recommend the ED scoring as Grade 0 (no signs of arthrosis), ED Grade 1 (mild dysplasia), ED Grade 2 (moderate dysplasia), and ED Grade 3 (severe dysplasia). Evidence of primary elbow disease, as well as the level of osteoarthritic findings, that is, UTN sclerosis and joint osteophytes, is used when determining the ED grade. A borderline sub-scoring between ED Grade 0 and ED Grade 1 is used in some countries (5, 7).

Sclerosis of the UTN is observed in the MCD (8–10). Its evaluation is based on direct visual assessment of bone radio-opacity and is considered to be a subjective parameter, and agreement in its assessment is positively correlated with the radiological experience of the observer (2). This subjectivity is compounded by images with different levels of radiographic exposure, different degrees of joint flexion or other changes associated with the dog's positioning, and variation between breeds. Recently, some studies have been carried out, which associate the increased image pixel values (radio-opacity) of UTN subchondral bone in digital images, evaluated by computerized image analysis, with clinical MCD in dogs (2, 11, 12). However, there are no studies exploring the computerized quantification of UTN sclerosis and relating the degree of sclerosis with ED grades.

The aims of the present study were to characterize the radiographic exposure of digital mediolateral elbow views and to measure the pixel value of the UTN subchondral bone in elbows classified as IEWG ED Grade 0, 1, 2, and 3, using a computer template. The hypothesis of our study was that there is a difference in pixel value of the UTN subchondral bone between ED Grade 0 elbows and dysplastic elbows (ED Grades 1, 2, and 3). The null hypothesis presumed no difference in opacity of the UTN subchondral bone between ED Grade 0 and dysplastic elbows.

## Materials and Methods

### Sample

This was a retrospective study based on the analysis of digital radiographs from the Danish Kennel Club (DKC) database for the ED screening scheme. Records and digital DICOM format flexed mediolateral images in the database for the period between 2012 and 2018 were available. From these records, dogs with a dysplasia-negative status (Grade 0) in the left limb were identified. From these dogs with normal left elbows, four groups of 20 dogs each, having a Grade 0, 1, 2, or 3 in the contralateral right elbow, were randomly selected. This process provided a study set of 80 dogs grouped according to ED grade (left elbow: right elbow) as follows: 0:0, 0:1, 0:2, and 0:3.

### Ethical Approval

The protocols were approved by the local Ethics and Administration Committee at the Department of Veterinary Clinical Sciences, University of Copenhagen, and performed in accordance with a Data Sharing Agreement between the DKC and the University of Copenhagen, which in turn complies with the requirements of the General Data Protection Requirements of the European Union.

### Computer Sclerosis Analysis

**Figure 1:**
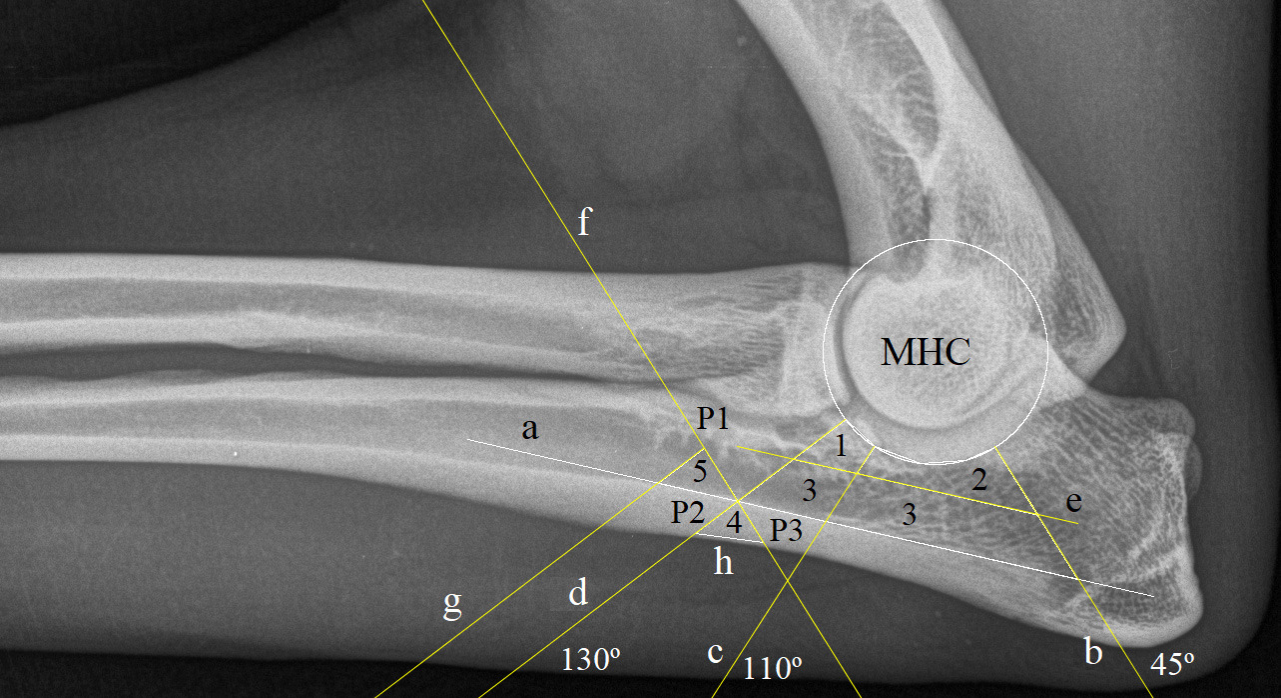
Regions of interest (ROIs) created in ImageJ: A sequence of instructions requiring some user input and performing some logical steps was created as a text file in the ImageJ programming language. For ImageJ, such a list is called a “macro.” Line “a” is drawn by the user as a tangent to the caudoproximal endosteal surface of the ulna, and three points are identified by mouse clicks on the edge of the medial humeral condyle (MHC). This user input allows for the automatic creation of the circular outline of the humeral condyle, together with three lines originating in the center of the MHC at angles of 45° “b,” 110° “c,” and 130° “d” to line “a,” a line “e” parallel to line “a” dividing the area bounded by lines “a,” “b,” and “d” and the humeral condylar, into two regions with areas in the ratio 1 (upper/cranial) to 2 (lower/caudal); line “f” is created again automatically by the macro, perpendicular to line “d” through the point of intersection between lines “d” and “a.” Finally, the user marks three points: P1 at the intersection of line “f” with the ulnar cranial endosteal cortex, and P2 and P3 at the points where lines “d” and “f” cross the periosteal caudal ulnar cortex, respectively. This allows the automatic creation of line “g,” which is parallel to line “d” and passes through P1, and line “h,” which joins P2 and P3. The macro then creates ROIs as follows: ROI-1, distal ulnar trochlear notch (UTN); ROI-2, middle UTN; ROI-3, caudal UTN (single area); ROI-4, cortical bone; ROI-5, bone marrow. The macro takes measurements from these five ROIs. The recorded measurements for each were mean, median, standard deviation, and area. The completed macro sequence thus creates lines and regions according to strict reproducible criteria.

Digital mediolateral elbow views were imported into ImageJ software (version 1.5.3 for Windows) and if necessary rotated so that the proximal radius was positioned horizontally in the image and the cranial part of the humerus orientated to the left (11). The range of pixel value in the images was normalized to 256 shades of gray (0, black; and 255, white) and displayed using a gray scale lookup table typical for radiography (radiolucent areas are relatively dark to areas that are more radiopaque) (11). The approach used was loosely based on published regions of interest (ROIs) of the region (11). This required a user initial input, and then the algorithm creates ROI-1, ROI-2, and ROI-3 for subtrochlear ulnar bone; ROI-4 for cortical bone; and ROI-5 for medullary regions of the ulna. Details of the input and of the created ROIs are shown in Figure 1. The macro used for this study and a sample image are included as Supplementary Material with the online version of this paper. Mean pixel intensity data from each ROI in the template were measured in ImageJ software and saved as a comma-separated values file.

For further analysis, the pixel intensity of each individual ROI-1, ROI-2, and ROI-3 was divided by the pixel intensity for ROI-4 in the same limb, in order to normalize the data. This step was taken into account for variation in radiographic exposure between images, as described previously (11). The ROI-5 was collected to study pixel intensity cortico-medullar differences in radiographic images. Dogs with evidence of UAP were not included in studies for evaluation of subtrochlear UTN pixel intensity.

**Figure 2:**
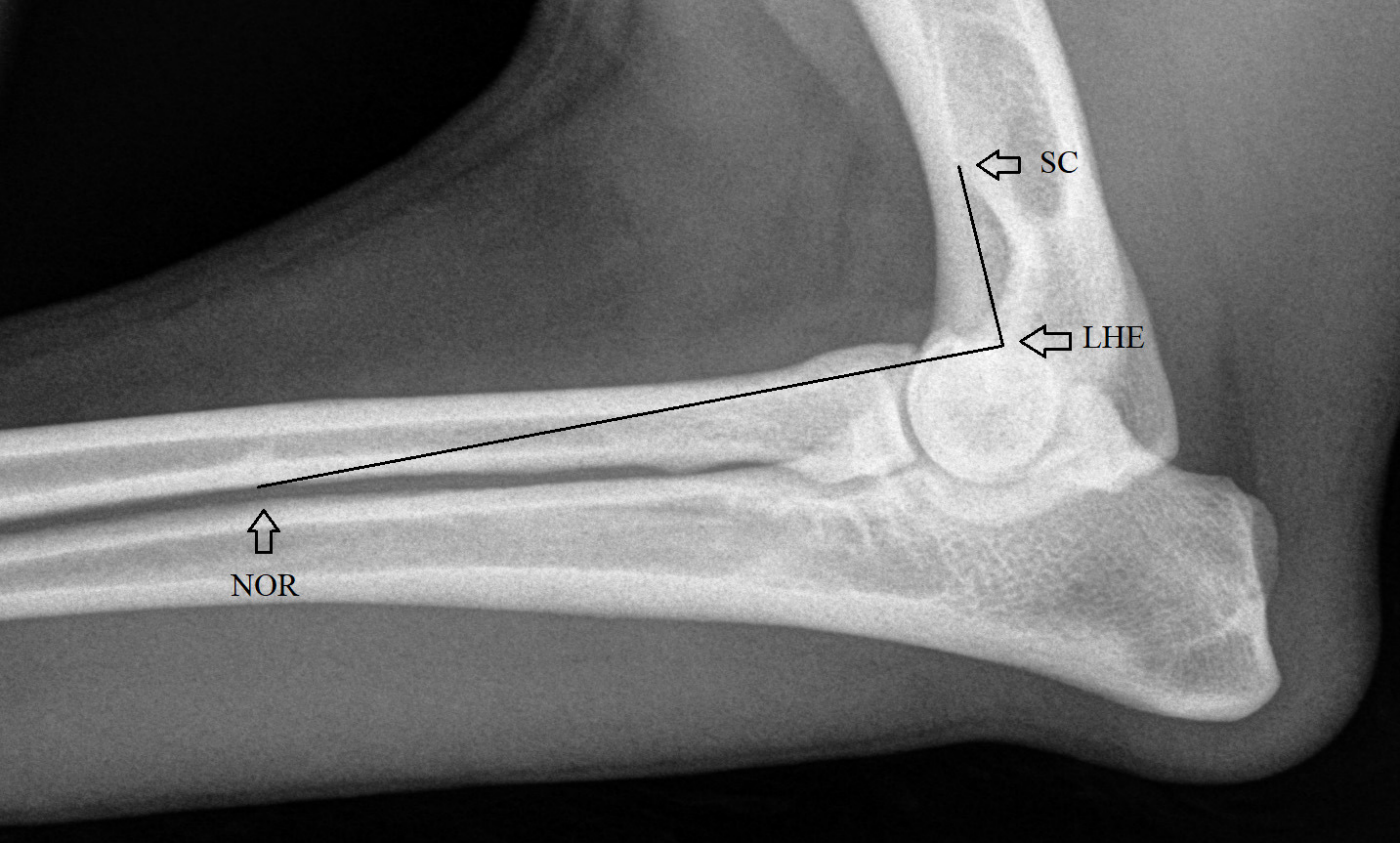
Anatomical landmarks used for measuring the elbow angles, the angular point in the lateral humeral epicondyle (LHE) (contact point of cranial border of medial epicondyle with the condyle) and the linking points at the nutrient orifice of the radius (NOR) and the intersection point of the lateral supracondylar crest and the cranial humeral endosteum (SC) (13).

The angles of mediolateral elbow views and the degrees of flexion were measured in the sample using a methodology previously described (13). Details of this measurement are shown in Figure 2.

### Statistical Analyses

Statistical analysis was performed on the raw and normalized pixel intensity in the different studied groups. The one-sample Kolmogorov–Smirnov test (OSKS) was used to evaluate the normal distribution of variables, and the one-way analysis of variance (ANOVA) and paired *t*-test were used to evaluate if normalized pixel intensity differed significantly between the studied groups. Scatterplot and boxplot graphical analyses were also performed for some of the data studied. The linear Pearson correlation was used to evaluate the association between elbow angles and some of raw and normalized pixel intensity variables. *P* < 0.05 was considered statistically significant. Statistical analysis was performed using the computer software SPSS (SPSS Statistics for Windows Version 27.0, IBM Corp., Armonk, NY, USA).

## Results

The age of the 80 dogs used in the sample ranged from 12 to 58 months, mean ± standard deviation (SD) 18.9 ± 9.6 months; and there were 36 males and 44 females. In the sample, there were animals of 22 different breeds; the most common was the German Shepherd, with 20 animals (25%).

**Figure 3:**
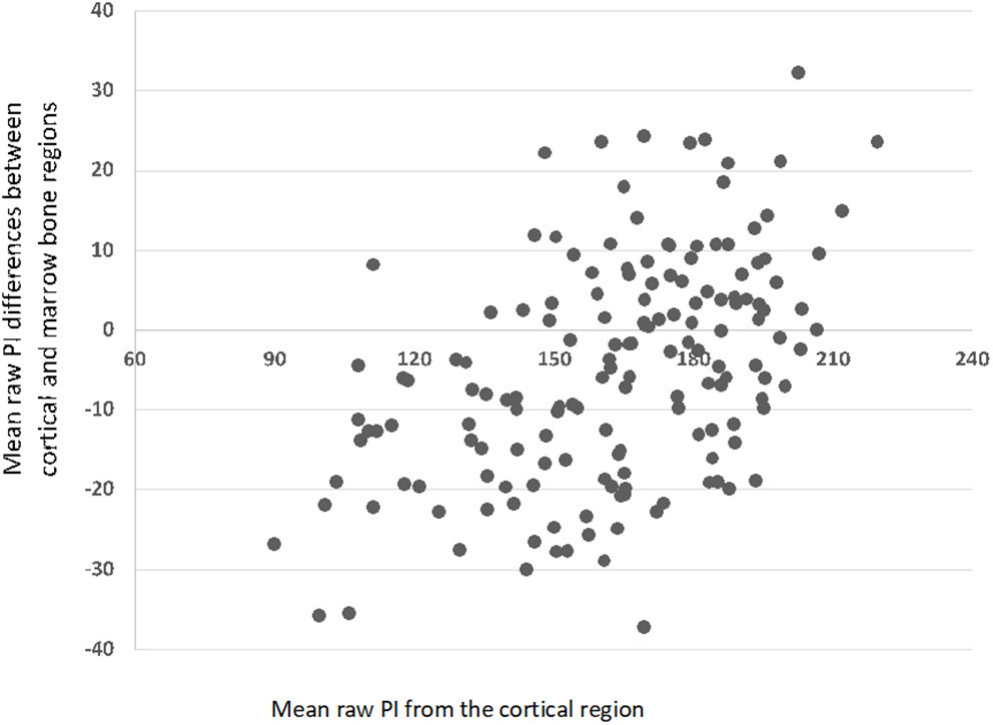
Scatterplot with mean pixel intensity (PI) differences between the cortical and marrow bone regions (*N* = 160).

**Figure 4:**
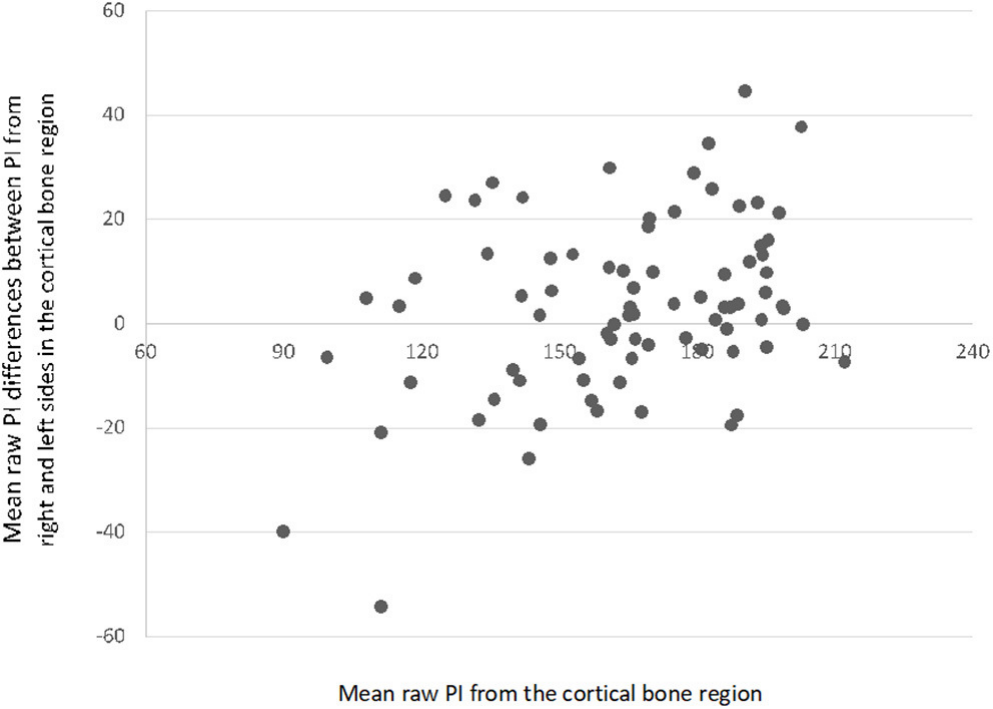
Scatterplot with mean differences between pixel intensity (PI) values from the right and left sides in the cortical bone region (*N* = 80).

**Figure 5:**
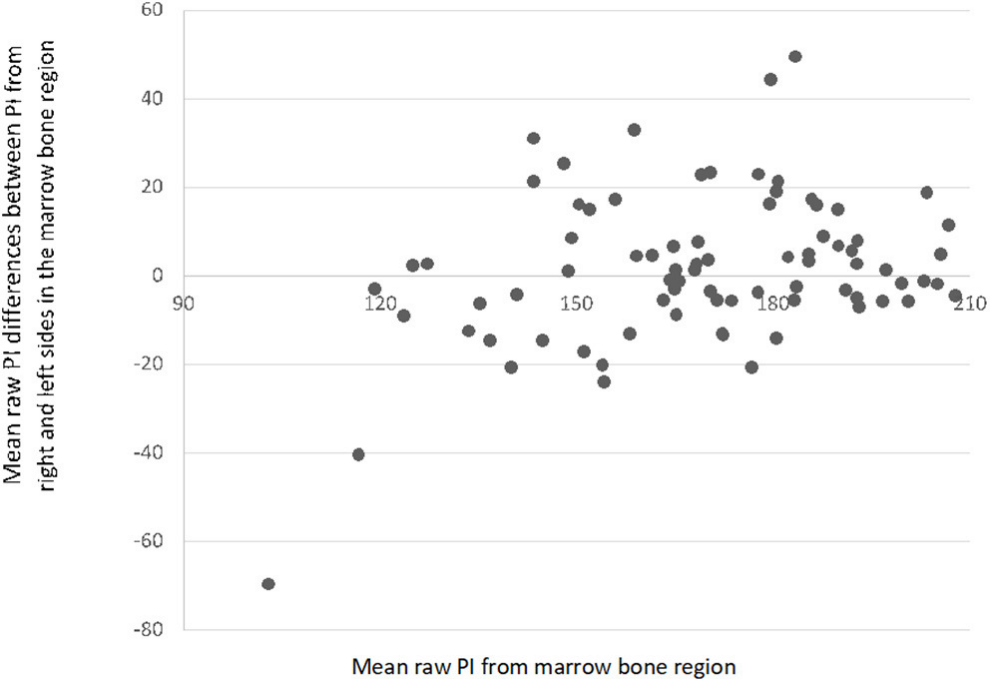
Scatterplot with mean differences between pixel intensity (PI) values from the right and left sides in the medullary region (*N* = 80).

The raw mean pixel intensity of cortical and bone marrow ROIs had a normal distribution (*P* > 0.05 in OSKS test). The raw mean pixel intensity of cortical ROI ranged from 90 to 220, mean ± SD, 163.1 ± 27.1; and the bone marrow ROI ranged from 103 to 212, mean ± SD, 168.1 ± 23.9. The differences in raw mean pixel intensity in the cortical minus bone marrow ROIs ranged from −37 to 32, mean ± SD, −5 ± 14.1 (Figure 3); those in the right minus left side cortical ROIs ranged from −44 to 45, mean ± SD, 3.2 ± 16.9 (Figure 4); and those in the right minus left side bone marrow ROIs ranged from −70 to 49, mean ± SD, 1.7 ± 17.1 (Figure 5).

Three dogs with UAP were excluded from ED Grade 3 group. The normalized mean pixel intensity of ROI-1, ROI-2, and ROI-3 also showed a normal distribution in the ED Grade (0, 1, 2, and 3) groups (*P* > 0.05). There was no statistically significant difference in normalized mean pixel intensity in the right ED Groups 0, 1, 2, and 3 in any subtrochlear UTN ROI-1, ROI-2, or 3 (*P* > 0.05, in ANOVA test) (Table 1).

**Table 1:** Normalized mean pixel intensity (PI) values in the different ulnar trochlear notch (UTN) regions of interest (ROIs) for the right-side elbow joints classified with different elbow dysplasia (ED) grades.

**UTN** **ROI**	**ED Grade**	**N**	**Mean PI values**	**Standard deviation**	**Standard error**	**95% Confidence interval for mean**		**Minimum** **value**	**Maximum** **value**	***P*-value** **ANOVA**
						**Lower bound**	**Upper bound**			
1	ED 0	20	1.11	0.11	0.03	1.06	1.16	0.91	1.33	0.55
	ED 1	20	1.18	0.25	0.06	1.07	1.30	1.00	2.01	
	ED 2	20	1.19	0.11	0.03	1.11	1.21	0.96	1.38	
	ED 3	17	1.16	0.12	0.03	1.1	1.22	1.02	1.50	
2	ED 0	20	0.97	0.09	0.02	0.93	1.01	0.82	1.09	0.14
	ED 1	20	1.00	0.15	0.03	0.93	1.07	0.83	1.49	
	ED 2	20	1.04	0.08	0.02	0.99	1.07	0.86	1.19	
	ED 3	17	1.03	0.09	0.02	1.0	1.08	0.94	1.27	
3	ED 0	20	0.91	0.07	0.01	0.88	0.94	0.78	1.05	0.25
	ED 1	20	0.89	0.11	0.03	0.84	0.94	0.66	1.16	
	ED 2	20	0.93	0.07	0.02	0.90	0.96	0.80	1.05	
	ED 3	17	0.94	0.07	0.02	0.90	0.98	0.82	1.10	

*ROI-1, distal UTN; ROI-2, middle UTN; ROI-3, caudal UTN*.

**Figure 6:**
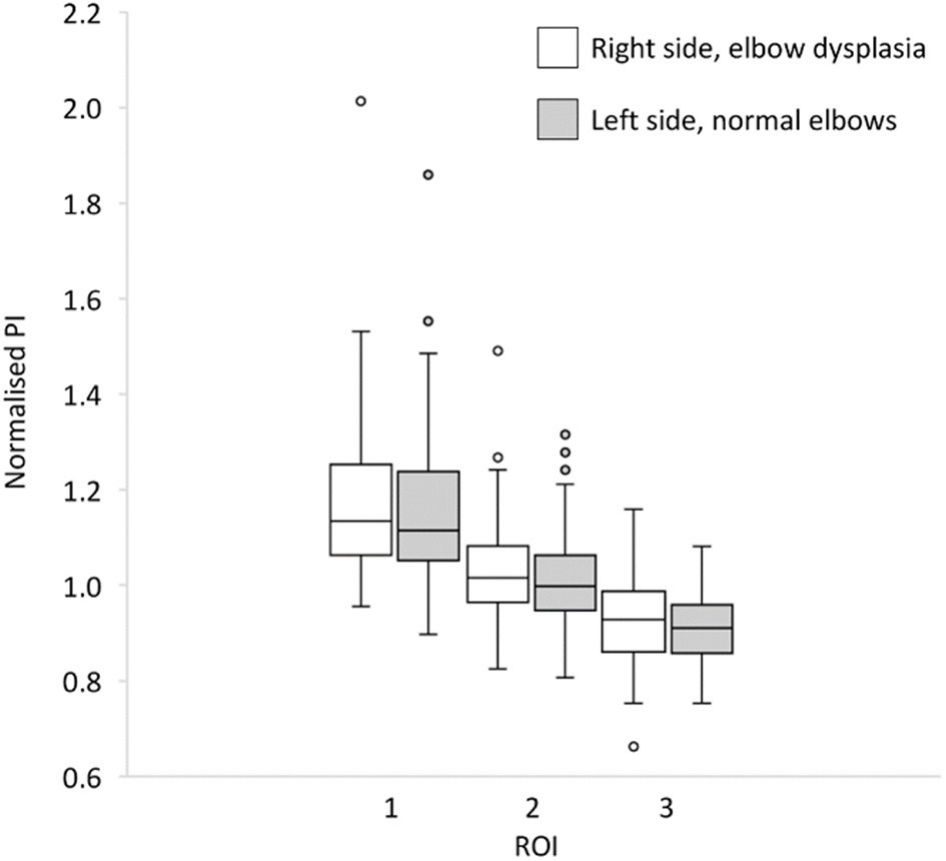
Box-and-whisker plot for comparison between normalized pixel intensity of ulnar trochlear notch (UTN) right-side elbows with elbow dysplasia and contralateral left side normal elbows on regions of interest (ROIs) 1 (distal UTN), 2 (middle UTN), and 3 (caudal UTN) (*N* = 60).

The mean pixel intensity of dysplastic right-side elbow joints (ED Grades 1, 2, and 3) were 1.17 ± 0.17, 1.02 ± 0.12, and 0.92 ± 0.09 for ROIs 1, 2, and 3, respectively. The corresponding values for the normal contralateral left joints were 1.14 ± 0.15, 1.00 ± 0.1, and 0.91 ± 0.08. There was a significant difference (*P* < 0.05 in paired *t*-test) in the normalized mean pixel intensity in dysplastic vs. non-dysplastic elbow joints for ROIs 1 and 2 (Figure 6).

The elbow angle in mediolateral views ranged from 22.1° to 97.4° (mean ± SD, 57.5° ± 16.6°) and showed some significant Pearson correlations with raw pixel intensity variables (Table 2). The association between subtrochlear UTN of raw and normalized pixel intensity ROIs 1, 2, and 3 was also statistically significant (*P* < 0.05) (Table 2).

**Table 2:** Pearson correlations between some of the studied variables: elbow angle (EA), raw (r) pixel intensity of subtrochlear regions of interest (ROIs) 1, 2, 3, 4 (cortex) and 5 (medulla), and normalized (n) subtrochlear nROI-1, 2, and 3.

	**rROI-1**	**rROI-2**	**rROI-3**	**rROI-4**	**rROI-5**	**nROI-1**	**nROI-2**	**nROI-3**
EA	0.24^*^	0.21^*^	0.21	0.17^*^	0.18^*^	0.00	0.04	0.10
rROI-1		0.92^*^	0.87^*^	0.71^*^	0.85^*^			
rROI-2			0.96^*^	0.83^*^	0.89^*^			
rROI-3				0.89^*^	0.90^*^			
rROI-4					0.86^*^			
nROI-1							0.85^*^	0.6^*^
nROI-2								0.84^*^

* *Significant correlation (P < 0.05)*.

## Discussion

The DKC ED screening scheme is based on the IEWG recommendations. The mediolateral elbow flexed view is sent by individual veterinarians to the DKC and ED is evaluated by a panel of scrutineers. Radiographs are scored as Grade 0 (no signs of ED) or Grade 1, 2, or 3 (dysplastic joints), depending on the analysis of the radiographic findings: level of UTN sclerosis, osteophyte size, or signs of primary elbow lesions. Our template was developed on ImageJ software in order to cover the entire UTN subchondral bone, without the overlapping of other bone structures, and to individualize the areas of interest, indicated in previous studies as the most suitable for detecting UTN sclerosis (2, 11, 12).

The random strategy of case selection was to try to eliminate some variability associated with the animal, since it allows the comparison between normal and ED in the same animal using the same imaging equipment and at the same time also allows a comparison between normal and ED in different animals. UAP cases were excluded from the subtrochlear pixel intensity evaluations because in UAP sclerosis is not a common feature and is not used for scoring purposes (14). The results allow us to accept the investigated hypothesis and exclude the null hypothesis for ROI-1 and ROI-2 (most distal and cranial regions of UTN), as the pixel intensity of the UTN subchondral bone in the dysplastic elbows was higher than that of the normal elbow (ED Grade 0) set of contralateral joints. Previous studies had already indicated these anatomic areas as the most predisposed regions for the evidence of UTN sclerosis (2, 11, 12). However, the differences in UTN radio-opacity registered in our research were very small, which did not allow us to recommend this methodology for clinical or ED scoring purposes. This fact is well-demonstrated by the ANOVA test, which did not show significant differences in the comparisons of radio-opacity on right joints scored as Grade 0, 1, 2, and 3 (Table 1). Other studies performing similar comparisons, but using only samples of animals affected with MCD and presenting clinical disease, showed evident and statistically significant differences in the subchondral bone UTN radio-opacity (10, 11). Both of these sample factors (MCD and clinical signs) will bias toward the presence of UTN sclerosis. Medial coronoid disease is a primary lesion associated with UTN distal area, as the base of the coronoid process contacts directly with the UTN. In the screening samples of ED control programs, most animals are asymptomatic, so the disease, if present, has not yet reached an advanced stage. Another factor that may have influenced our results is that some ED primary conditions (OCD and incongruency) may promote less subchondral UTN sclerosis than MCD. A previous study indicated that UTN sclerosis may even be reduced in elbow joints affected only with UAP (14).

The bone density distribution in the subchondral bone plate of the elbow joint of dogs was described using computed tomographic osteoabsorptiometry in normal elbow joints (15). Lower ulnar bone density at the apex of the medial coronoid process with high UTN sclerosis in case of MCD was also described (16). An age-dependent increase in subchondral bone density at the elbow joint, including the UTN, was observed (14, 15). We did not compare the mean pixel intensity between animals with different ages, body weight, conformations, or breeds. We normalized our data to ROIs within the same patient, so any such effects would have been masked.

Our results showed that the evaluation of UTN sclerosis in digital radiographs may become more difficult than in conventional radiographs, since images are actually very different in terms of general radiographic density and contrast. The range of pixel values of cortical and bone marrow among the sample was very large; and sometimes, the mean pixel intensity of the bone marrow ROI-5 was greater than that of the cortical ROI-4 (Figure 3). These facts may be related to some breed variability in cortical and bone density, to different parameters used for the radiographic beam (X-ray tube current and voltage, exposure time, and focus–detector distance) during the acquisition of radiographs, but also due to differences in the image processing software used from different manufacturers (17). Even in film radiography, it is established that high kilovoltage techniques will reduce image contrast, and this was likely a cause of variation in our study also (Figure 3).

The differences between cortical and bone marrow pixel intensity observed in the contralateral views (right vs. left) are interesting and unexpected (Figures 4, 5). It is likely that imaging software versions, hardware, and beam factors will be the same for contralateral elbows in the same dog. It is accepted however that digital image processing can be influenced by the contents of the field of view, how much of the detector plate is exposed, and the amount and type of tissue and other objects (such as positioning markers), which are included in addition to the target anatomical area. These effects however should be random between the right and left elbows in our study and thus do not explain the difference in pixel intensity seen between the diseased right and normal left elbows. The difference we did see is however unlikely to be clinically useful since ED is often bilateral, and one cannot assume that one will have a normal contralateral limb for comparison.

An additional variability in this study arises from the ED screening images themselves. The elbow angle on the mediolateral views in our sample varied from 22.1° to 97.4° and together with different limb rotations could have interfered with our analysis of UTN sclerosis due to some radiographic summation with bone and soft tissues joint structures. Our results indicate that larger elbow angles are positively associated with more opacity in all raw pixel intensity ROIs (1, 2, 3, 4, and 5) due to soft tissue overlap and opacity summation effects. However, the absence of an elbow flexion angle association with the normalized variables seems to indicate that the interference is eliminated when data normalization is performed (Table 2). The significant correlation between subtrochlear ROIs 1, 2, and 3 are expected results, taking into account the anatomical proximity between these regions.

The radiographic image has many optical illusions (18), and one of them is well-demonstrated in this study when assessing cortical bone opacity. Cortical bone may appear to have a uniform and higher opacity than the marrow to the human eye, but in reality, it is non-uniform, with greater bone opacity toward the endosteal surface when compared with the exosteal region. The apparent higher cortical exosteal opacity is due to the optical effects of surrounding lower attenuating soft tissue opacity. Our template evaluates the cortical opacity with a triangular ROI, with its base on the external cortical. It is likely that mean cortical pixel intensity would have been higher had we chosen an ROI that included a more endosteal cortical bone.

Despite the difficulties encountered in identifying ulnar notch sclerosis in this study using objective analysis of digital radiographs, it remains well-established that bone sclerosis is a feature of ED. The mechanism underlying the development of sclerosis is unknown, despite its importance as indicator in the radiographic diagnosis of MCD. It is thought to occur most likely as a result of superimposition of periarticular osteophytosis and an increase in subchondral bone mineral density (10). However, Lau et al. (10) demonstrated the sclerosis of the subchondral UTN bone without interference of periarticular osteophytosis by comparing ulnas with and without sclerosis by computed tomography and radiography. The sclerosis is characterized by the loss of trabecular bone architecture and increased radiographic density. In ED, UTN sclerosis has been linked to increased stiffness of subchondral bone and higher vulnerability of articular cartilage to injury (19). Previous studies concluded that there is a statistically significant association between UTN sclerosis and medial coronoid disease (2, 6, 8, 12), especially when sclerosis is localized in the more distal part of the UTN (11). Quantification of bone density of the medial coronoid process in sound dogs and dogs with fragmented coronoid process (FCP) has also been reported (8).

## Conclusions

The pixel intensity of the UTN subchondral bone in the dysplastic elbow joints was higher compared with that in the normal contralateral elbow joints (ED Grade 0). However, these differences may be more evident if MCD were a feature of ED group. Elbow joint extension is associated with a higher radio-opacity in elbow joint area; however, the normalization of data eliminated this effect in the subchondral UTN region. Digital radiographs are associated with many variabilities due to radiographic parameters and image processing algorithms. These variabilities in pixel intensity make computer quantification of radiographic bone opacity of the UTN in ED difficult and problematic. For visual assessment, it is possible that human image evaluator is prompted by other features, possibly related to trabecular pattern rather than absolute pixel intensity, when concluding on the presence of “bone sclerosis.” Further studies are needed to study computer-assisted UTN pixel intensity evaluation using as gold standard not the human visual evaluation but other more accurate tools, like the computed tomography trabecular bone architecture.

## Data Availability Statement

The raw data supporting the conclusions of this article will be made available by the authors, without undue reservation.

## Author Contributions

AV: acquisition of data and drafting of the manuscript. SA-P: drafting of the manuscript and critical revision of manuscript. MG: contribution to concept/design, data analysis/interpretation, drafting of the manuscript, and critical revision of manuscript. FM and DN: contribution to concept/design, data analysis/interpretation, and critical revision of manuscript. All authors contributed to the article and approved the submitted version.

## Conflict of Interest

The authors declare that the research was conducted in the absence of any commercial or financial relationships that could be construed as a potential conflict of interest.
