# Supplementary material for: Digital Analysis of Subtrochlear Sclerosis in Elbows Submitted for Dysplasia Screening
Source: Front Vet Sci. 2021 May 12;8:664532. doi: 10.3389/fvets.2021.664532 (PMC8149609; doi:10.3389/fvets.2021.664532)
Supplement: Supplementary file 1 [file Data_Sheet_1.zip › read_me.rtf]

This file and the accompanying ImageJ macro and JPEG image,  are supplementary material to the paper “Digital analysis of subtrochlear sclerosis in elbows submitted for dysplasia screening.”
 Authors: Ana Válega, Sofia Alves Pimenta, Dorte H. Nielsen, Fintan J. McEvoy and Mário Ginja. 
(Frontiers in Veterinary Science (2021)).


To run the ImageJ macro described in that paper: 

1. Install ImageJ ( available at "https://imagej.net/Fiji"). This will create a directory called Fiji.app at the location of your choose.
2. Copy the file "UlnarNotchAnalysis.ijm" to the directory "Fiji.app/macros"
3. Open ImageJ
4. Open the image file "elbow.jpg" in ImageJ
5. In ImageJ select the menu item "Plugins" and the submenu "Macros/Install"
6. Select the macro "UlnarNotchAnalysis.ijm" from the "macros" directory and click open.
7. Select again the menu item "Plugins/Macros", and then "UlnarNotchAnalysis" which is now available at the bottom of the drop-down menu.


These steps will install and start the ImageJ Macro.  As an alternative one can open both the Image and the Macro file via the ImageJ - File/Open menu. The ImageJ text editor will show the content of the macro. Selecting "run" in the text editor to execute the macro.

Once running the macro will prompt the user to draw a line and to mark certain points (6 in all) on the image.  These are detailed in the accompanying paper.

The macro will output a comma separated file containing ROI name and measurements.  This file is saved to the same directory as the image to which it refers.
