# Supplementary figures and images for: Digital Analysis of Subtrochlear Sclerosis in Elbows Submitted for Dysplasia Screening
Source: Front Vet Sci. 2021 May 12;8:664532. doi: 10.3389/fvets.2021.664532 (PMC8149609; doi:10.3389/fvets.2021.664532)

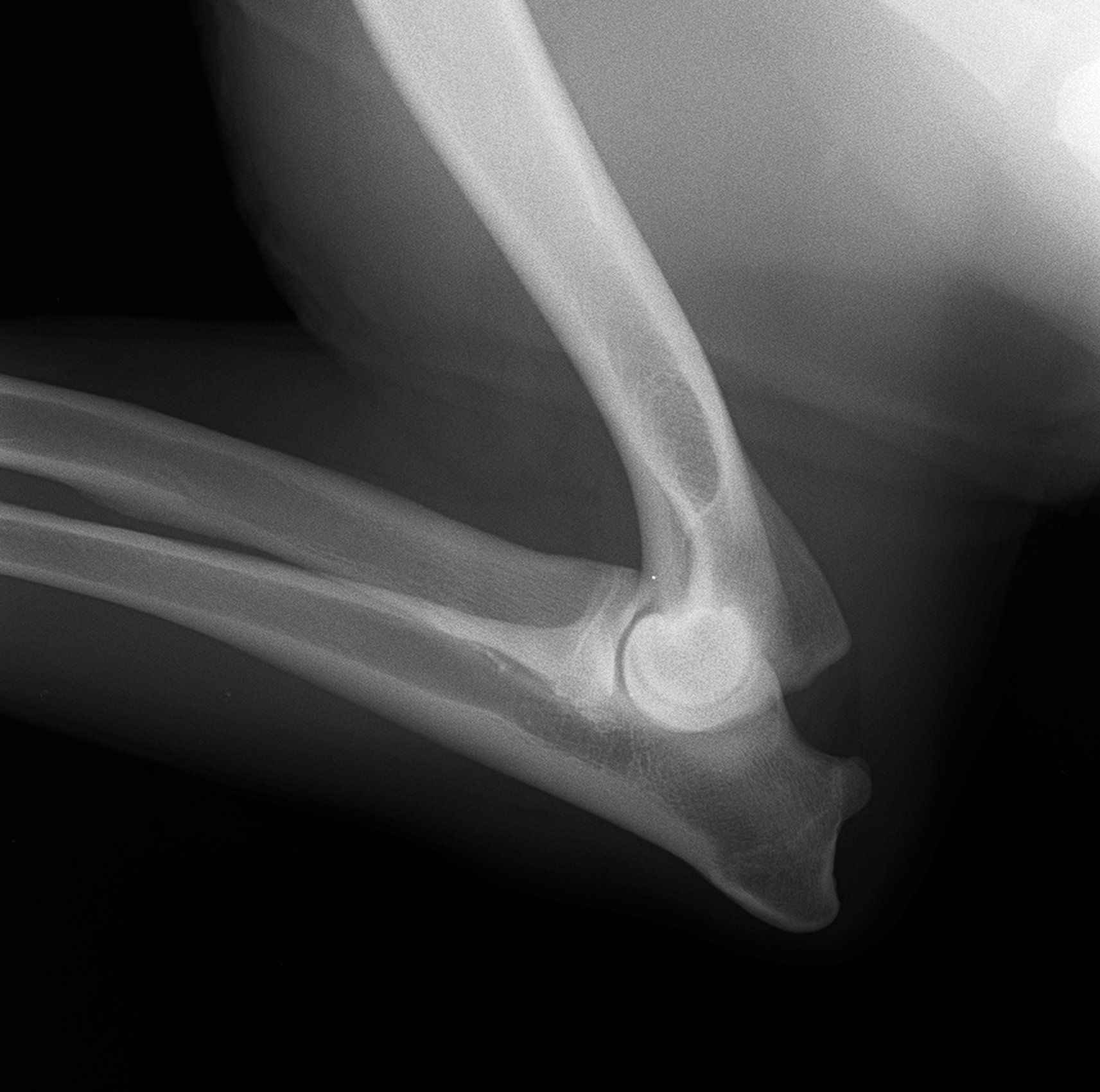

Supplement: Supplementary file 1 [file Data_Sheet_1.zip › elbow.jpg]
